# Isolation and Purification of Fucoxanthin from Brown Seaweed *Sargassum horneri* Using Open ODS Column Chromatography and Ethanol Precipitation

**DOI:** 10.3390/molecules26133777

**Published:** 2021-06-22

**Authors:** Yuemei Ye, Jingwen Sun, Liting Wang, Junwang Zhu, Wei Cui, Hongyan Hou, Jinrong Zhang, Chengxu Zhou, Xiaojun Yan

**Affiliations:** 1 College of Food and Pharmaceutical Sciences, Ningbo University, Ningbo 315211, China; yeyuemei717@163.com (Y.Y.); 17863993693@163.com (J.S.); wangliting200@163.com (L.W.); zhujunwang520@163.com (J.Z.); houhongyan1909@163.com (H.H.); 2 Ningbo Key Laboratory of Behavioral Neuroscience, Zhejiang Provincial Key Laboratory of Pathophysiology, School of Medicine, Ningbo University, Ningbo 315211, China; cuiwei@nbu.edu.cn; 3 Key Laboratory of Applied Marine Biotechnology of Ministry of Education, Ningbo University, Ningbo 315211, China

**Keywords:** fucoxanthin, ODS column chromatography, ethanol precipitation, purification, brown seaweed

## Abstract

As an abundant marine xanthophyll, fucoxanthin (FX) exhibits a broad range of biological activities. The preparation of high-purity FX is in great demand, however, most of the available methods require organic solvents which cannot meet the green chemistry standard. In the present study, a simple and efficient purification approach for the purification of FX from the brown seaweed *Sargassum horneri* was carried out. The FX-rich ethanol extract was isolated by octadecylsilyl (ODS) column chromatography using ethanol–water solvent as a gradient eluent. The overwhelming majority of FX was successfully eluted by the ethanol–water mixture (9:1, *v*/*v*), with a recovery rate of 95.36%. A parametric study was performed to optimize the aqueous ethanol precipitation process by investigating the effects on the purity and recovery of FX. Under the optimal conditions, the purity of FX was 91.07%, and the recovery rate was 74.98%. Collectively, the eco-friendly method was cost-efficient for the purification of FX. The developed method provides a potential approach for the large-scale production of fucoxanthin from the brown seaweed *Sargassum horneri.*

## 1. Introduction

As one of the most abundant marine carotenoids, fucoxanthin (FX) accounts for approximately 10% of the total production of carotenoids in nature [1]. FX is naturally distributed in diverse marine organisms, especially brown seaweeds and microalgae [2,3,4,5]. FX possesses a unique structure, including an unusual allenic bond, a 5,6-monoepoxide, and a conjugated carbonyl group in the polyene chain of the molecule [6]. However, such a unique structure is unstable and can be easily isomerized or degraded by heating, aerial exposure, and illumination due to its poor chemical stability during the process of extraction, purification, and storage [7,8,9]. FX exhibits broad biological activities, including antioxidant, anti-inflammatory, anti-obesity, anti-cancer, anti-diabetic, anti-malarial, anti-senile dementia, and anti-angiogenic properties [4,10]. Moreover, FX has been declared safe for consumption by humans or animals [11]. In general, healthy supplements with FX are recognized as safe by the European Food Safety Authority, Japan’s Food for Specified Health Uses, and the US Food and Drug Administration [11].

In recent years, FX has been investigated for a wide range of applications in the food, pharmaceutical, and cosmeceutical industries due to its broad healthcare functions [1,9,12]. The demand for FX is now emerging in the global market. The global market size of FX production (the content is about 1%) was approximately 500 tons in 2015, and it is expected to increase by at least 5.3% per year between 2016 and 2021 [11]. Moreover, it is expected to increase to USD 120 million by 2022 [12].

Owing to the broad application prospects, great progress has been made in the preparation of FX with various methods. However, due to the complexity and low efficiency of the chemical synthesis for FX, FX production on an industrial scale is still confronted with this challenge [12]. At present, FX is mainly extracted and purified in large-scale production from diverse brown seaweeds due to their relatively low cost [13]. Various approaches have been developed for the purification of FX, including centrifugal partition chromatography [14], silica gel column chromatography (SGCC) [12], preparative thin-layer chromatography (PTLC) [15], high-performance liquid chromatography (HPLC) [16], and high-speed countercurrent chromatography [17]. However, the high price is still the major problem that restricts the application of fucoxanthin because these technologies are difficult to use on a large scale for the industrial purification of FX due to the high cost, low efficiency, complex processes, and time requirements [18]. Most importantly, most of the available methods for the preparation of high-purity fucoxanthin require organic solvents which cannot meet the green chemistry standard. Therefore, it is necessary to develop novel cost-effective purification technologies for the development of a new nutraceutical industry.

The brown seaweed *Sargassum horneri*, an economically important edible brown seaweed, is widely distributed in East Asia, including China, Korea, and Japan [19,20]. In recent years, *S. horneri* has been found to contain a great deal of bioactive secondary metabolites, especially FX [21]. Moreover, *S. horneri* extracts exhibit various biological activities, such as anti-inflammatory [22], reverse passive cutaneous anaphylaxis [23], antioxidant [24], and anti-cancer properties [25]. *S. horneri* can be considered as a valuable FX-producing algal candidate due to its high content of FX [26,27].

Currently, ODS open column chromatography is widely used for the purification of many compounds. For example, high-purity DHA was successfully purified by an industrial high-performance liquid chromatography method using two columns packed with octadecylsilica (reverse-phase ODS) [28]. As a common purification method, precipitation has been widely used in the purification of bioactive secondary metabolites, such as oligogalacturonic acids and polysaccharides, from various natural organisms [29,30]. Moreover, as an environmentally sustainable process, ethanol precipitation has several distinct advantages, including simple operation, ease of scaling up preparation, solvent safety, and green products [31].

In the present study, an eco-friendly and efficient purification approach of FX from the brown seaweed *S. horneri* was developed by using a combination of octadecylsilyl (ODS) column chromatography (CC) and ethanol precipitation. The new purification method has many advantages, such as being environmentally friendly, ease of scaling up, low cost, and green products with high yield and purity.

## 2. Results and Discussion

### 2.1. Isolation of FX by ODS Column Chromatography

**Figure 1:**
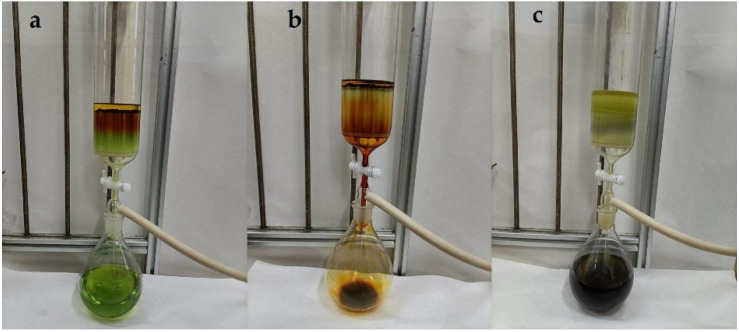
Fractionation process of the crude extract from *Sargassum horneri* with octadecylsilyl (ODS) column chromatography. (**a**) Addition of ethanol:water (7:3, *v*/*v*), (**b**) addition of ethanol:water (9:1, *v*/*v*), (**c**) addition of absolute ethanol.

**Figure 2:**
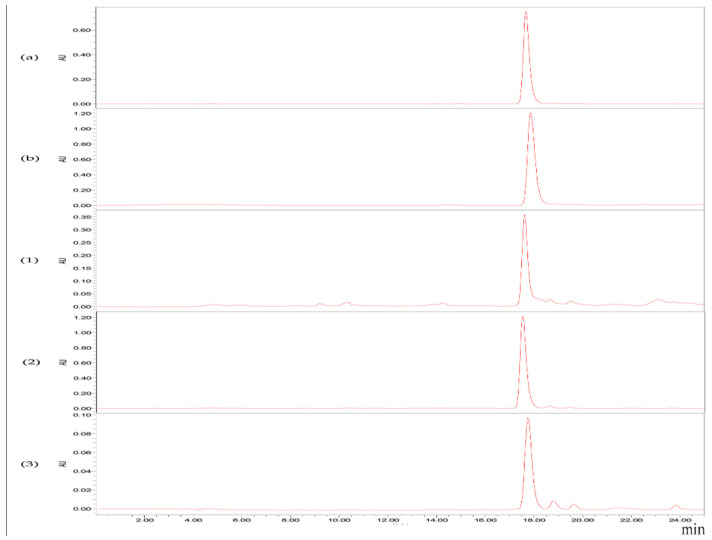
HPLC analysis of samples taken throughout the fractions obtained during the ODS chromatographic purification with three-step gradient (70%, 90%, and 100% in ethanol:water, *v*/*v*). (**a**) Fucoxanthin (FX) standard solution with a concentration of 100 mg/L; (**b**) the *S. horneri* extract solution with an FX concentration of 139.84 mg/L; (**1**) the fraction eluted by ethanol:water (7:3, *v*/*v*); (**2**) the 2-fold diluted fraction eluted by ethanol:water (9:1, *v*/*v*); (**3**) the fraction eluted by absolute ethanol.

**Figure 3:**
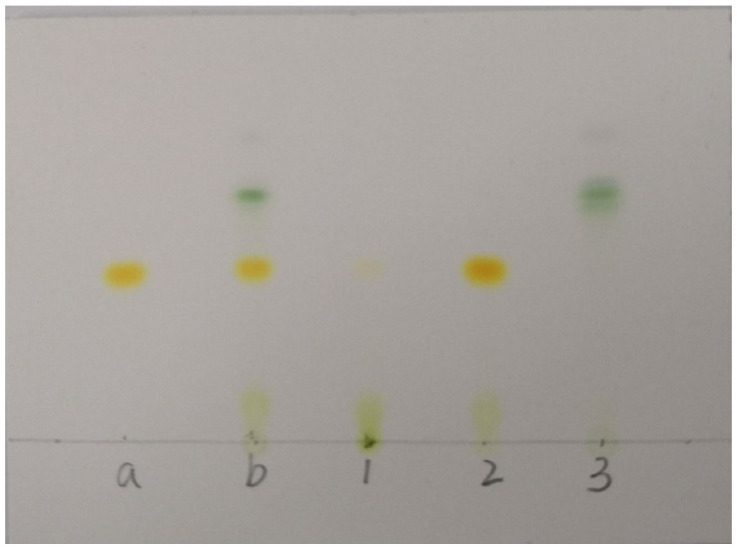
TLC analysis of samples taken throughout the fractions obtained during the ODS chromatographic purification with three-step gradient (70%, 90%, and 100% in ethanol:water, *v*/*v*). Sample a, FX standard; Sample b, the crude *S. horneri* extract; Sample 1, the fraction eluted by ethanol:water (7:3, *v*/*v*); Sample 2, the fraction eluted by ethanol:water (9:1, *v*/*v*); Sample 3, the fraction eluted by absolute ethanol.

Figure 1 illustrates the isolation process of the crude extract from the seaweed *S. horneri* using ODS open tubular column chromatography. The obtained fractions were analyzed using HPLC (Figure 2) and TLC (Figure 3). Figure 3 shows the TLC analysis results, showing that green polar impurities were effectively eluted by the ethanol–water mixture (7:3, *v*/*v*). However, the HPLC analysis results showed that the recovery rate of FX was 0.86%. The results were further verified by the TLC analysis (Figure 3, sample 1). It should be noted here that the overwhelming majority of FX was successfully eluted by an ethanol–water mixture (9:1, *v*/*v*), and the recovery rate of FX in the FX-rich fraction was 95.36%. Moreover, the TLC analysis (Figure 3, sample 2) showed that minor impurities were co-eluted with FX. In this study, for the first time, FX was successfully isolated from brown seaweed by ODS column chromatography. The successful case of the purification of DHA was essentially practical, and will provide a working basis for the purification of fucoxanthin at an industrial scale [28]. Ethanol is a green chemical used in a variety of pharmaceutical and chemical industries. Additionally, most ethanol can be recycled in industry in several ways, such as distillation [32].

### 2.2. Effects of Vacuum Evaporation on FX-Rich Fraction

**Figure 4:**
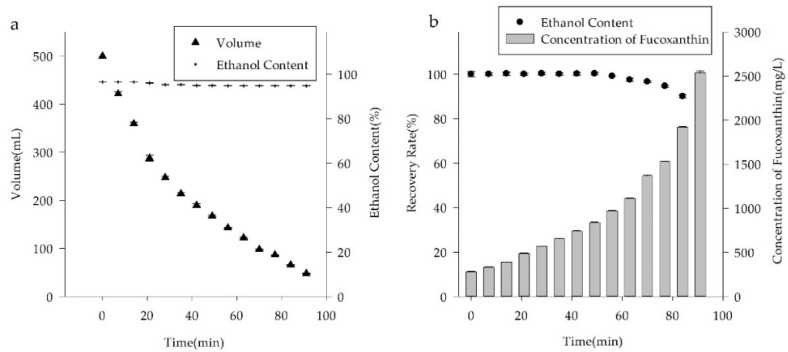
The variations of four key parameters during the vacuum evaporation process. (**a**) The volume and ethanol content of the concentrated solution. (**b**) The concentration and recovery rate of FX in the concentrated solution.

In the present study, we studied the variations in four parameters of the concentrated solutions during a vacuum evaporation procedure for the FX-rich fraction. Figure 4 shows that during the vacuum evaporation process, the volume of the concentrated solution was gradually decreased, whereas the content of FX in the concentrated solution was rapidly increased. When a 500 mL FX-rich fraction with an FX concentration of 280 mg/L was concentrated to 50 mL using a vacuum evaporator at 298 K, the concentration of FX in the concentrated solution became 2535.85 mg/L. Notably, the ethanol content in the concentrated solution was about 95% throughout the evaporation procedure. The FX-rich fraction exhibited a special azeotropic phenomenon. The ethanol content of the FX-rich fraction (ethanol:water = 9:1, *v*/*v*), was very similar to that of an ethanol/water azeotrope solution (ethanol:water = 95.63: 4.37, g/g) [33]. In addition, during the evaporation procedure, FX was still completely dissolved because its concentration in the solution did not reach the saturation concentration of the solvent system. In the present study, the purification of FX by the vacuum evaporation method was not feasible.

According to the literature, FX is extremely prone to isomerization or degradation when heated or exposed to air and light [7]. In the present study, the recovery rate of FX was negatively correlated with the evaporation time. When a 500 mL FX-rich fraction was concentrated to 50 mL using a vacuum evaporator at 298 K, the recovery rate of FX in the concentrated solution was 86.33%.

### 2.3. Efficiency of Ethanol Precipitation in the Purification of FX

**Figure 5:**
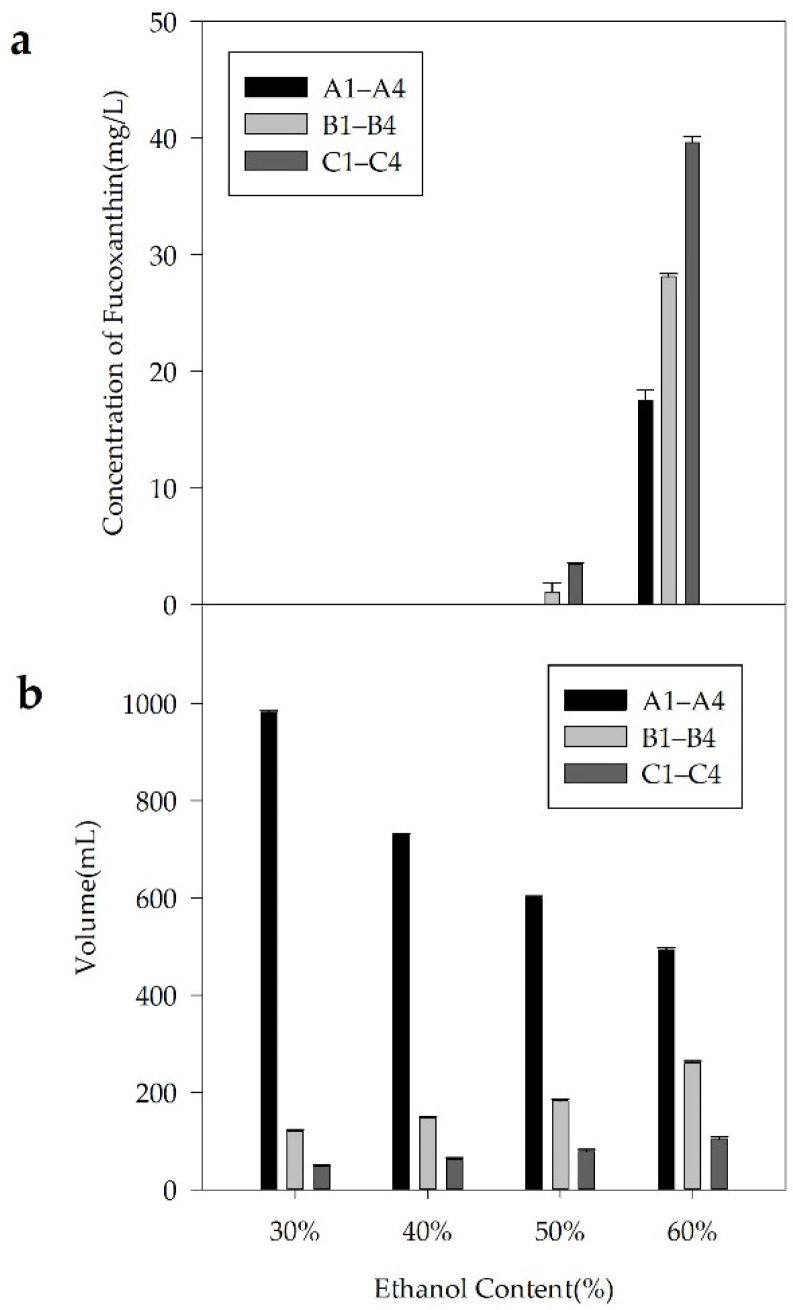
The FX concentration (**a**) and volume (**b**) of the solutions A1–A4, B1–B4, and C1–C4.

**Figure 6:**
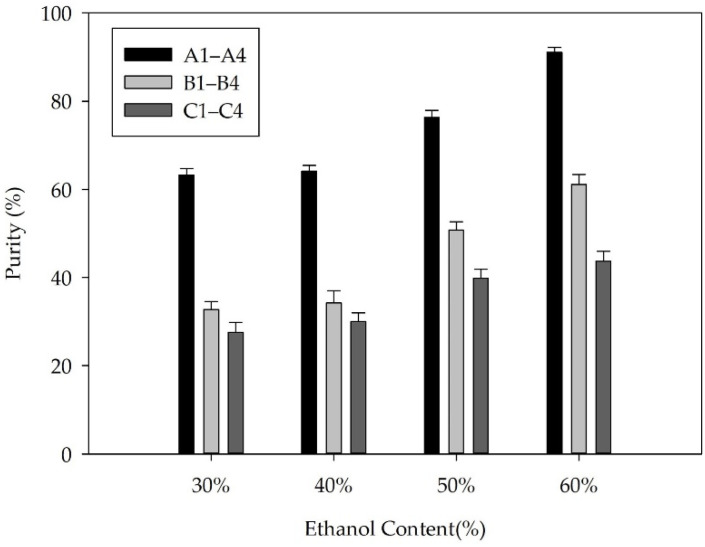
Purity of FX obtained from the solutions A1–A4, B1–B4, and C1–C4.

**Figure 7:**
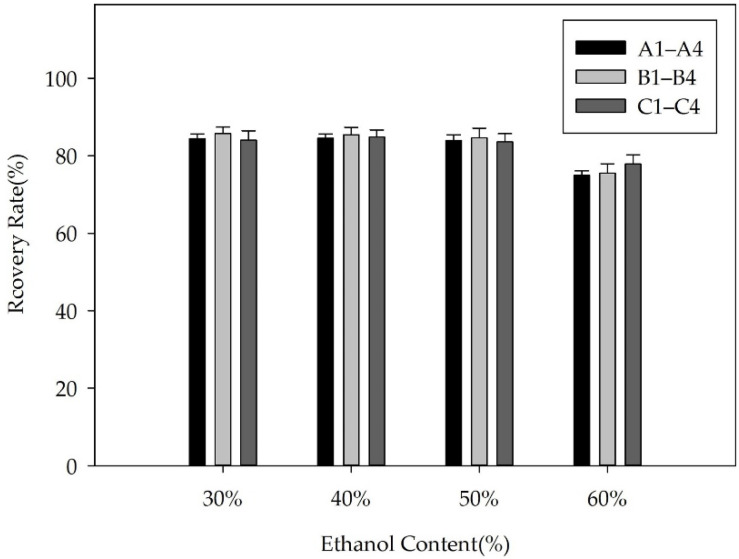
The recovery rate of FX obtained from the solutions A1–A4, B1–B4, and C1–C4.

**Figure 8:**
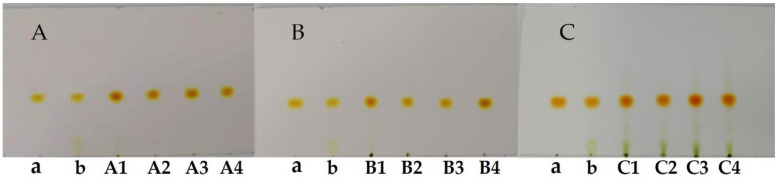
TLC results of the obtained FX solid. Sample a, FX standard; Sample b, the *S. horneri* extract; (**A**) samples A1–A4 were derived from the solutions A1–A4, respectively; (**B**) samples B1–B4 were derived from the solutions B1–B4, respectively; (**C**) samples C1–C4 were derived from the solutions C1–C4, respectively.

The precipitation of FX could be achieved by adjusting the ethanol content in the solution. In the present study, we investigated two patterns to control the ethanol proportion in the solution. Figure 5 shows the FX concentration in the 12 solutions, including solutions A1–A4, B1–B4, and C1–C4, as well as the volume of the solutions. The purity, recovery, and TLC results of the freeze-dried FX purified by ethanol precipitation are shown in Figure 6, Figure 7 and Figure 8, respectively.

The recovery rate of FX obtained from the three solutions A4, B4, and C4 was 74.98%, 75.50%, and 77.91%, respectively. For the remaining solutions, the recovery rate of purified FX was more than 83%. The results demonstrated that part of FX was dissolved in the supernatants containing 60% ethanol (Figure 5a), resulting in the corresponding loss of FX recovery. Generally, the solubility of lipids increases with increasing ethanol content, which shows that properly increasing the ethanol concentration of the supernatant is beneficial to remove more fat-soluble impurities. Additionally, the fat-soluble impurities co-eluted with FX by the ODS CC might lead to a synergistic effect, resulting in significant differences in FX content among the solutions of A4, B4, and C4 (Figure 5a) [34]. (2) The recovery rate of FX was not notably affected by the volume of solutions. In the present study, the volume of solutions A1, B1, and C1 was significantly different (Figure 5b), whereas there was no significant difference in the recovery rate of FX (Figure 7). The result was mainly attributed to the poor concentration of FX in the supernatant. (3) FX is prone to degradation when exposed to air and light [7]. In the present study, the recovery rate of FX from the 12 solutions was more than 74% during the sample purification. In this study, adding water directly to 60% ethanol content was suitable for effective precipitation of FX from the FX-rich fraction due to the higher product purity and saving of energy. Under the optimal precipitation conditions, the purity of FX was 91.07%, and the recovery rate was 74.98%.

### 2.4. Identification of FX

**Figure 9:**
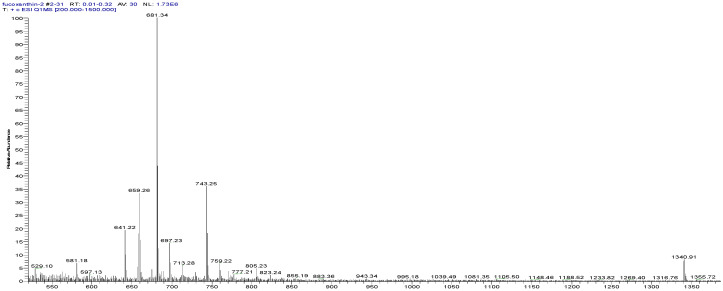
The positive ESIMS spectrum of FX obtained from the solution A4.

**Figure 10:**
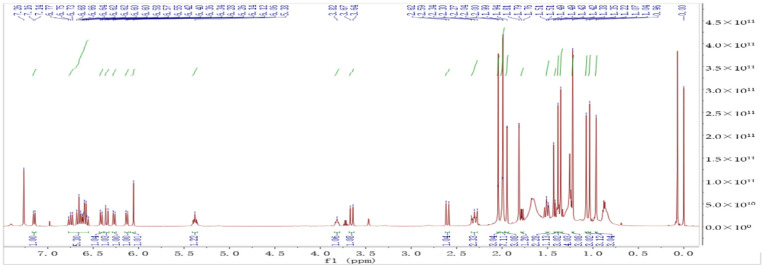
^1^H-NMR (600 MHz, CDCl_3_) spectrum of FX obtained from the solution A4.

The *S. horneri* extract was analyzed by HPLC to detect FX. The molecular mass of the refined product was proposed as FX based on the fragment patterns at *m*/*z* 659.26 and 581.34, which correspond to [M + H]+ and [M + Na]+, respectively (Figure 9). The refined product was subjected to 1D nuclear magnetic resonance (NMR) spectroscopy (Figure 10) and identified as all-trans FX according to the published literature [35] among the naturally occurring geometrical isomers of FX [36].

## 3. Materials and Methods

### 3.1. Materials

All organic solvents used for TLC and column chromatography were of analytical grade and purchased from Huadong Chemicals (Hangzhou, China). Chromatographic-grade solvents used for HPLC analysis were obtained from Anpel Laboratory Technologies (Shanghai, China). FX standard (purity ≥ 95%, HPLC) was supplied by Sigma-Aldrich Co., Ltd. (Shanghai, China). ODS silica gels (ODS, S-50 μm) were purchased from YMC Co., Ltd. (Tokyo, Japan). The silica gel prefabricated GF254 plate used for TLC analysis was obtained from Qingdao Marine Chemical Factory (Qingdao, China). The brown seaweed *S. horneri* was cultured and harvested from the regions near Gouqi Island (30°42′ N, 122°46′ E) in Zhejiang Province, the People’s Republic of China.

### 3.2. Apparatus

Column chromatography separation was performed using an ODS silica gel open tubular column in the stepwise elution mode. HPLC analysis was conducted on a Waters Alliance 2695 HPLC system, equipped with a quaternary solvent delivery system, an autosampler, a 2996 diode array detector (Waters, Milford, MA, USA), and a 250 mm × 4.6 mm i.d., 3 µm, YMC C-30 carotenoid column (Waters Co., Ireland). Freeze drying was carried out using a laboratory freeze dryer (Scientz^®^Scientz-10N, freeze dryer, Ningbo Scientz Biotechnology Co., Ltd., Ningbo, China)

### 3.3. FX-Containing Crude Preparation

In this study, the concentration of fucoxanthin was 0.28 mg/g in fresh *S. horneri*. The overall yield of FX was 18.43 mg/100 g wet weight from the fresh *S. horneri.* FX was extracted from the fresh brown seaweed *S. horneri* via a series of steps, including solvent extraction, low-temperature concentration, and freeze drying. Briefly, FX extraction was conducted by a maceration extraction system under the following conditions: 80% (*v*/*v*) ethanol as solvent, solid to solvent ratio of 1:3 (*w*/*v*) for 2 h at 298 K. Then, the extracted solution was concentrated until the ethanol content reached 30% in the concentrated solution using a vacuum evaporator (IKA^®^RV8 Rotary Evaporators, Staufen, Germany) at 298 K. Then, fat-soluble substances including FX, precipitated out from the concentrated solution, were collected by filtration through a 600-mesh screen. The precipitate was washed with water at a solvent to solid ratio of 10:1 (*v*/*w*) and centrifuged at 4500 rpm for 10.0 min. The wet FX-containing crude was subjected to a freeze-drying process (Scientz^®^Scientz-10N, freeze dryer, Ningbo Scientz Biotechnology Co., Ltd., Ningbo, PR China). Finally, with the extraction procedure, the FX-containing crude extract with a purity of 8.74% was obtained, and the recovery rate was 65.82%.

### 3.4. ODS Column Chromatographic Experiments

FX was further isolated from the FX-containing crude extract using an ODS open tubular column in stepwise elution mode. Briefly, the FX-rich extract from the brown seaweed *S. horneri* was loaded onto an open preparative chromatography column (30 cm × 7.5 cm i.d.) containing ODS (50 μm, dry weight of 140 g) and eluted at a flow rate of 1 mL/s with a gradient mode of an ethanol–water mixture (7:3, 9:1, 10:0, *v*/*v*). After the fraction (approximately 700 mL) eluted by the ethanol–water mixture (7:3, *v*/*v*) was discarded, a 2800 mL FX-rich fraction eluted by ethanol–water mixture (9:1, *v*/*v*) was collected. The residue was eluted by absolute ethanol. The fractions were collected and examined by TLC and HPLC. The amounts of eluted FX were determined, and the corresponding yields were calculated.

### 3.5. Vacuum Evaporation of FX-Rich Fraction

To evaluate the effectiveness of vacuum evaporation, a 500 mL FX-rich fraction with an FX concentration of 280 mg/L, which was obtained by the ODS open tubular column chromatography, was concentrated to 1/10 of the original volume using a vacuum evaporator (IKA^®^RV8 Rotary Evaporator) at 298 K. The volume and ethanol content of the concentrated solution were measured every 7 min during the concentration process (Figure 4), as well as the concentration and recovery rate of FX in the concentrated solution.

### 3.6. Purification of FX by Precipitation

**Figure 11:**
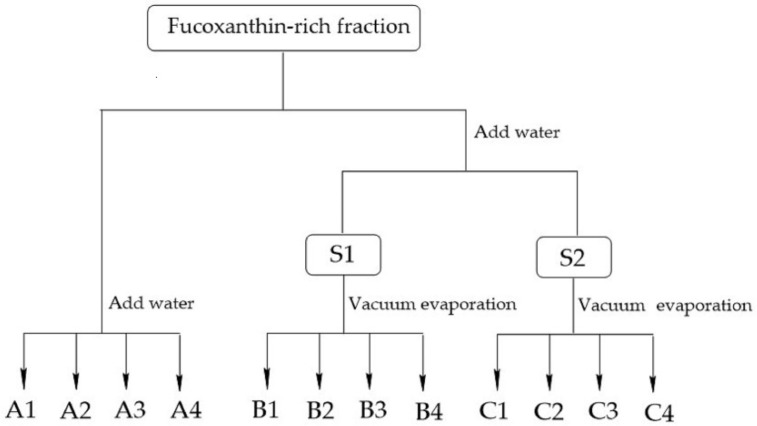
Optimization of the precipitation of FX. Solution S1 was prepared by adding water to 70% ethanol content, and solution S2 was prepared by adding water to 80% ethanol content. The solutions A1–A4 were prepared by adding water directly. The solutions B1–B4 and C1–C4 were prepared by vacuum evaporation at room temperature. The ethanol content in group 1 solutions (including A1, B1, and C1), group 2 solutions (including A2, B2, and C2), group 3 solutions (including A3, B3, and C3), and group 4 solutions (including A4, B4, and C4) was 30, 40, 50, and 60%, respectively. All operations were performed at room temperature.

To optimize the precipitation conditions of FX, 12 FX-rich fractions (300 mL of each) with an FX concentration of 280 mg/L were divided into three groups. Then, the ethanol content of the fractions was adjusted to certain proportions, respectively. The detailed adjustment method is shown in Figure 11. To assure the recovery of FX, the optional solutions obtained by the adjustment method were kept at room temperature for 48 h. Subsequently, FX-rich solids were precipitated from the solutions and collected by centrifugation at 4500 rpm for 10.0 min. They were washed with water at a solvent to solid ratio of 10:1 (*v*/*w*) and centrifuged at 9500 rpm for 10.0 min to completely recover FX. The water-washing process was repeated three times to completely remove water-soluble impurities. The wet refined FX was subjected to a freeze-drying process (Scientz^®^Scientz-10N, freeze dryer, Ningbo Scientz Biotechnology Co., Ltd., Ningbo, PR China) and then sealed.

### 3.7. Characterization

The HPLC analysis of FX was carried out using the method described by Kim et al. with minor modifications [37]. All solutions were filtered by a 0.45 μm organic filter membrane before analysis. The purified FX was subjected to HPLC using a Waters Alliance 2695 HPLC system, equipped with a quaternary solvent delivery system, an autosampler, and a 2996 diode array detector (Waters, Milford, MA, USA). The HPLC conditions were as follows. A YMC C-30 carotenoid column (250 × 4.6 mm ID, 3 μm particle size, Waters, Ireland) was used for the separation. A methanol and water solvent system was used as the mobile phase at a flow rate of 0.7 mL/min at a column temperature of 35 °C. The solvent gradient program was as follows. Methanol/water ratio was increased from 90:10 to 100:0 over 20 min, and then 100% methanol was held for the next 5 min. A DAD within a range of 200–800 nm was used, and the chromatogram was recorded at 450 nm. To measure the concentration of FX, the FX standard was dissolved in methanol to prepare eight different standard solutions with a concentration range of 1–200 μg/mL. The standard solutions were used to construct the calibration curve, and the linearity of the curve was indicated by the corresponding correlation coefficient. A calibration curve of FX was constructed within a range of 1–200 μg/mL. The mean linear regression equation was Y = 156285X − 181164 with a correlation coefficient of 0.9994, where Y is the peak HPLC area for FX and X is the concentration of FX. An excellent correlation existed between the peak area and concentration of the FX.

The TLC method was used to evaluate the effectiveness of isolation and purification of FX. Spots of the samples were applied on the TLC plates using a capillary syringe. A developing reagent, with a petroleum ether (boiling point 60~90 °C) to acetone ratio of 2:1 (*v*/*v*), was added to the glass vessel for a few minutes to ensure the saturation of solvent vapor inside the vessel. After drying the spots, the TLC plates were transferred to the glass vessel, and the chemical substances were developed by the developing solvent. Once the reagent reached the preset position, the plates were taken out of the vessel and observed under visible light. Finally, the sample spots were photographed immediately for documentation and visual analysis.

ESI-MS analyses were performed using electrospray ionization–quadrupole–time of flight mass spectrometry (ESI-Q-TOF MS, Waters, Milford, CT, USA). ^1^H-NMR spectra were recorded using an AVANCE 600 MHz NMR spectrometer at 298 K with TMS as an internal standard (Bruker, Fällanden, Switzerland).

### 3.8. Statistical Analysis

All experiments were performed in triplicate. All data were expressed as mean ± SD. Analysis of variance (ANOVA) followed by LSD was used for statistical comparisons. Levels of *p* < 0.05 were considered to be statistically significant. All statistical analyses were processed using the IBM SPSS 26 package.

## 4. Conclusions

Brown seaweeds are significant sources of FX with broad biological activities. Significant progress has been made in the isolation and purification of FX from brown seaweeds and/or microalgae over the past 20 years. A novel eco-friendly and cost-efficient purification approach of FX from the brown seaweed *S. horneri* was carried out in our current work. To preserve the safety of the obtained FX, ethanol was used as the extraction, elution, and precipitation material in our study. The fresh *S. horneri* was extracted with ethanol, which was easy to operate on an industrial scale. After freeze-drying, the FX-rich extract was isolated by ODS column chromatography using ethanol–water solvents as gradient eluents. An overwhelming majority of FX was successfully eluted by the ethanol–water mixture (9:1 *v*/*v*), with a recovery rate of 95.36%. The FX-rich fraction was treated with the ethanol–water mixture containing 30–60% ethanol for the further precipitation of FX. Different patterns of adjustment were adopted to adjust the ethanol content for FX precipitation. Under the optimal precipitation conditions, the purity of FX was 91.07%, and the recovery rate was 74.98%. The purity of FX was assessed by HPLC, and the structure of the purified FX was confirmed with NMR and MS. The successful application of ODS column chromatography in the industrial purification of DHA could imply the feasibility of FX purified by ODS column chromatography at an industrial scale. Although a great amount of ethanol was consumed during the course of ODS column chromatography, the overwhelming majority of ethanol could be recycled in industry in several ways to avoid high costs and environmental pollution. In addition, the ethanol precipitation method is a common purification method, and has been widely used in the pharmaceutical industry. This new method was cost-effective, easy to scale up, and eco-friendly. The obtained FX with high purity was safe and reliable. Collectively, the abovementioned results suggested that the method could be used and further improved to obtain purified FX from fresh *S. horneri.*

## Data Availability

Data available on request.
